# Automated Morphologic Differentiation Between Iron Deficiency Anemia and Thalassemia

**DOI:** 10.1002/jcla.70097

**Published:** 2025-09-03

**Authors:** Julien Guy, Marie‐C Béné, Ramon Simon Lopez, Marc Maynadié, Céline Row

**Affiliations:** ^1^ Hematology Biology Dijon University Hospital Dijon France; ^2^ Hematology Nantes University Nantes France; ^3^ Mindray Scientific Research Zug Switzerland

**Keywords:** AI‐aided morphology, red blood cells, target cells

## Abstract

**Background:**

Iron deficiency anemia (IDA) and hemoglobinopathies (HbP) are two frequent conditions characterized by microcytemia. Published criteria/scores discriminating these conditions with hematology analyzer parameters are not fully satisfactory. Although patients with HbP have been reported to have more red blood cells (RBC) with a target cell (TC) morphology than patients with IDA, obtaining TC percentages remains a time‐consuming task since at least 1000 RBC must be examined.

**Methods:**

Using the Mindray CAL 8000 2.0 0111 and MC‐80 module, 152 microcytic samples from 51 patients with IDA and 101 with HbP were analyzed. Data from RBC parameters used in published scores were collected, as well as the percentages of target cells automatically provided by the MC‐80.

**Results:**

Patients with IDA had significantly lower median hemoglobin level, red blood cell numbers, and mean corpuscular hemoglobin concentration than those with HbP, yet had more microcytes. Using TC percentages provided by the MC‐80 module, receiving operator characteristic curves identified this parameter as the most discriminant to segregate patients with IDA or HbP. With a 0.4% threshold, this yielded a 74.2% sensitivity and 86.3% specificity, confirming that patients with HbP have significantly higher TC percentages.

**Conclusion:**

The automated identification and enumeration of abnormal RBC performed by Mindray MC‐80 rapidly provides TC percentages, allowing for a fast discrimination between IDA and HbP in samples with microcytosis, orienting early towards confirmatory tests for these disorders. Moreover, this study confirms TC, which can obviously be obtained through other methods, as a robust parameter in this context.

## Graphical abstract

Discrimination between iron‐deficiency anemia (IDA) and hemoglobinopathies (HbP). Identification of microcytemia by the hematology analyzer. Automated launch of smear preparation and staining. Artificial Intelligence‐aided identification and evaluation of the percentage of target cells. Discriminative threshold 0.4%.

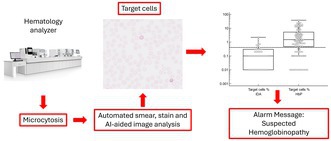

## 1 Introduction

Iron deficiency anemia (IDA) is the most common form of anemia in the world. Induced by a deficit in dietary iron, impaired iron intestinal absorption, or iron loss due to chronic bleeding, it is reported to affect 30% of women of reproductive age, 37% of pregnant women, and 40% of children [1]. Because of the insufficient iron available, the heme to globin ratio is lower than normal in erythroblasts, which results in one or two more divisions than normal during erythropoiesis, inducing microcytosis and hypochromia in red blood cells (RBC) lacking normal levels of hemoglobin (Hb) [2]. Anomalies in Hb production can also result from faulty globin proteins, as seen in the anemia of hemoglobinopathies (HbP), also leading to microcytosis [3].

In the routine laboratory, hematology analyzers have been devised to generate alarms when several parameters of a complete blood count (CBC) suggest anemia. Examination of the results may then inform the investigator about the severity of Hb decreased level, microcytosis, macrocytosis, or normocytosis, as well as other RBC parameters. Yet, further investigations are then needed to discriminate between IDA and HbP [4].

Over the years, many indexes, based on CBC results, have been proposed to perform this discrimination. In a meta‐analysis of 99 publications reporting on several thousand patients, Hoffmann et al. [5] retained 12 such indexes as performing best. The parameters used in these publications were the RBC count, mean corpuscular volume (MCV), mean corpuscular hemoglobin concentration (MCHC), red cell distribution width (RDW) and microcytosis/hypochromia in different combinations. However, great heterogeneity was found in the performance of these indexes, not really linked to analyzers, but more, and unexpectedly, due to geographical areas. Several publications also proposed different cut‐offs than those initially published. All in all, these literature searches identified the three best methods for adults as those reported by Green and King [6], using MCV, RDW, and Hb; Bessman et al. [7], based on RDW; and England and Fraser [8], using MCV, RBC count, and Hb. For children, the indexes devised by Sirdah [9] and Eshani [10] were considered to perform best. In a series of pregnant women, the Metzner index was also found useful to discriminate between IDA and HbP [11]. The most recent of such indicators, the CRUISE index, was developed in 2019 [12]. Of note, all of these need to collect information from the CBC and apply formulas.

Morphologic assessment to differentiate IDA and hemoglobinopathies could be another, more straightforward discriminating tool, but has been the object of sparse reports. The presence of target cells (TC), RBC with a central spot of Hb surrounded by a clearer ring, itself delimited by an external ring of Hb, has been described long ago [13] and reported to be more frequent in hemoglobinopathies than IDA. In 2008, Harrington et al. [14] reported a consistent study attempting to differentiate these two diseases and anemia of chronic diseases (ACD). Prekeratocytes (precursors of spiculated cells) and pencil cells were the two RBC morphologies that allowed for the best differentiation, yet with a rather large overlap and sensitivities around 70% or below. TC were seen in this study in 95% of patients with IDA and in 93% of beta‐thalassemia cases, but their numbers were very different and useful to rule out ACD (1 vs. 16.9 per 1000 RBC in IDA and 12.4 in beta‐thalassemia) but not to discriminate between IDA and HbP. However, recommendations by the Internal Council for Standardization in Hematology (ICSH), less than 10 years ago, reckoned the variability of microscopy evaluation owing to the ability and skill of the operator [15]. Moreover, these recommendations require that at least 1000 RBC are counted, which is not only time‐consuming but also suffers from subjectivity and lack of documentation. Additionally, the low incidence of TC increases variability as is the case for all counts of rare cells [16].

There thus remains an unmet need for a rapid, straightforward indication of the etiologic assessment of a fortuitously detected microcytic anemia. Here, the recent combination of Mindray CAL 8000 2.0 0111, an automated hematology analyzer comprising a blood cell analyzer (BC‐6800‐Plus), the SC‐120 slide maker‐stainer, together with the MC‐80 digital morphology module and the LabXpert integrated software, was used to explore how this combination could automatically provide discriminative parameters between IDA and HbP, using RBC parameters and automatic enumeration of TC.

## 2 Patients

From June 14, 2023 to December 29, 2023, 152 microcytic blood samples, defined by a MCV < 80 fL assessed with a routine hematology analyzer, were selected for re‐evaluation. Fifty‐one were from patients with IDA defined as having a low ferritin level (≤ 8 μg/L). They were 37 women and 14 men, with a median age of 37 years (interquartile range [IQR] 23–48). The 101 others (48 women, 53 men, median age 36 years, IQR 20–55) were from known patients with various types of HbP, confirmed by Hb electrophoresis (Table 1). Of note, only these two conditions were considered and samples with microcytosis for other reasons (vitamin B6 or copper deficiency, heme synthesis defect or inflammatory anemia) were not retained. The samples had been sent to the laboratory for routine CBC and no specific bloodletting was performed, making patient consent and ethics board authorization unnecessary, as per French law. Moreover, patient data were anonymized, only identified by a sample number without access to patient identity.

**TABLE 1:** Sample numbers and characteristics.

Final diagnosis	*Number*
IDA	51
β‐thalassemia minor	28
β‐thalassemia major	2
HbC heterozygosity	3
HbC homozygosity	1
HbE heterozygosity	8
HbE disease	2
D‐Punjab heterozygosity	1
Sickle‐cell α+/β + −thalassemia	25
Sickle‐cell disease or sickle‐cell β0‐thalassemia	28
Sickle‐hemoglobin C disease	3
Total	**152**

## 3 Methods

All samples were re‐analyzed with the Mindray CAL 8000 2.00111 and MC‐80 module. RBC parameters were detected based on red cell enumeration, Hb quantification, and cell size using impedance technology. These are combined by the instrument to provide the classical criteria of MCV and MCHC. Other red cell parameters, obtained from the reticulocyte channel, inform about Hb red cell content, erythroblasts, and reticulocytes cell fluorescence using a nuclear dye, and further appreciate red cell size using laser light scatter at different angles. Abnormal values in these parameters trigger alarms through the LabXpert integrated software.

Besides, the indexes reported by Sirdah [9] and Ehsani [10] were evaluated as well as the M/H ratio [17, 18] dividing the percentage of microcytes by that of hypochromic cells. These tests were chosen because they had been reported as providing the best sensitivity and specificity values [5].

**FIGURE 1:**
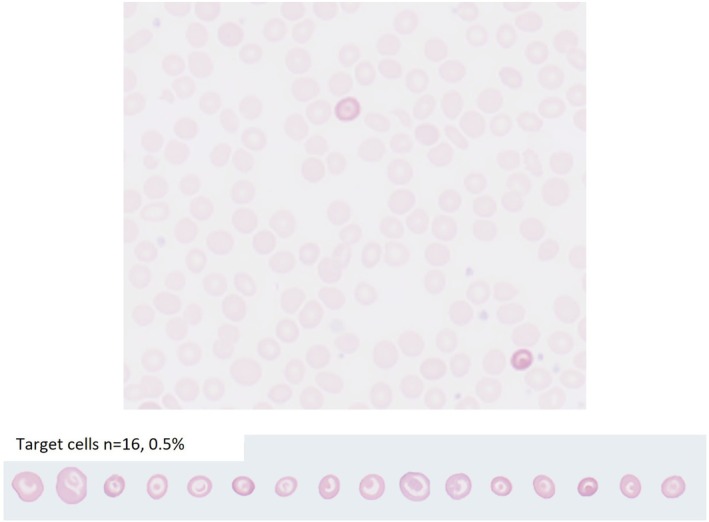
Result of the morphological identification of target cells by MC‐80. The top image shows how target cells are highlighted on demand (here 2 target cells). The bottom image shows the result of MC‐80 analysis, in this case 16 cells were observed for a percentage of 0.5%.

Additionally, blood smears from these samples were performed using the SC‐120 instrument, included in the CAL 8000 automation line, and analyzed by the MC‐80 automated AI‐aided morphology assessment module, able to identify and quantify target cells among other types of red cells. The instrument captures and displays with high throughput (1 min total reading time) high‐quality images using an immersion oil objective of the appropriate reading region of the smear where cells are properly separated. TC percentages were provided automatically by the AI morphology module trained to recognize and enumerate abnormal RBC as well as displaying them (Figure S1). It can also, on demand, highlight them on any area of the slide displayed on the computer screen together with numeral data. Here focus was brought to TC and microcytes (Figure 1). At least 2000 RBC are analyzed for TC counting (Figure S1), but they can be visualized over the whole scanned area through the software display.

Statistical analyses to compare groups were performed with the Mann Whitney‐*U* test. Results are expressed as medians and IQR. Receiver operator characteristics (ROC) curves were used to determine thresholds, sensitivity, and specificity of discriminating parameters. These tests used MedCalc (Ostend, Belgium) software.

## 4 Results

### 4.1 Classical Parameter Differences Between Patients With IDA or HbP

IDA patients had a significantly lower median Hb levels, at 9.8 g/dL (IQR 8.7–10.9), than patients with HbP, at 11.0 g/dL (IQR 9.8–11.9, *p* = 0.0002). This was also the case for RBC numbers at 4.3 × 10^12^/L (IQR 3.9–4.6) vs. 4.8 × 10^12^/L (IQR 4.03–5.38, *p* = 0.0047) and MCHC at 31.4 g/dL (IQR 30.4–32.0) vs. 32.9 g/dL (IQR 32.3–33.9, *p* < 0.0001) yet with often broad overlap. MCV was comparable at 74.8 fL (IQR 69.5–77.3) vs. 71.4 fL (IQR 64.8–79.1, *p* = 0.745). IDA patients had more microcytes, at 13% (IQR 5.5–21) vs. 7.7% (IQR 4.5–12.7, *p* = 0.0034 Figure S2).

### 4.2 MC‐80 Automatic Assessment of Target Cell Percentages

**FIGURE 2:**
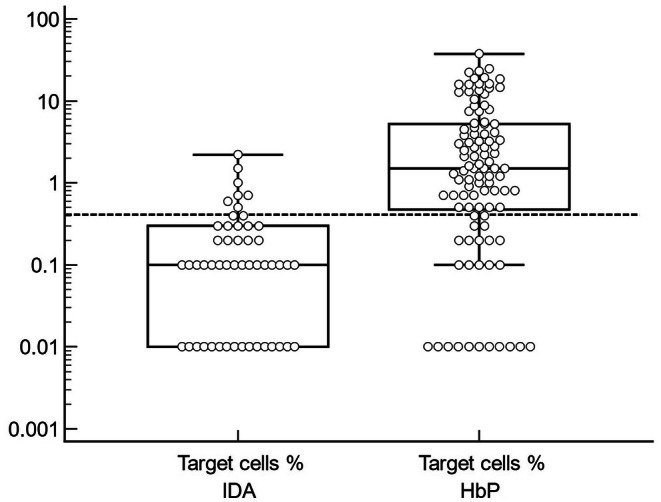
Discrimination between IDA (*n* = 51) and HbP (*n* = 101) based on the percentage of target cells identified by the MC‐80. The dotted line indicates the segregating 0.4% threshold. The ordinate presents percentages on a logarithmic scale. Box‐whisker graphs indicate the median and 25/75 percentiles. Student *t‐*test confirmed a statistically significant difference with *p* < 0.0001.

TC automatically identified and quantified by the MC‐80 provided a median of 0.1% (IQR 0.01%–0.3%) for patients with IDA vs. 1.5% (IQR 0.47–5.22) for patients with HbP (*p* < 0.0001), with almost no overlap (Figure 2). This parameter yielded the most significant ROC curve with an area under the curve (AUC) of 0.85 (*p* < 0.001) yielding 74.2% sensitivity and 86.3% specificity for target cells at a threshold of 0.4%. Of note, considering only β‐thalassemia minor patients versus IDA, the AUC for a 0.6% threshold reached a 0.882 value with 82.1% sensitivity and 90.2% specificity. The other classical indices devised to differentiate IDA and HbP provided less significant results (Table 2 and Figure S3).

**TABLE 2:** Thresholds and ROC curves results in decreasing specificity, and with the best threshold to differentiate IDA from β‐Thalassemia (see also Figure S3).

Parameter	Criterion threshold	AUC	Sensitivity	Specificity
Discrimination between iron deficiency anemia and hemoglobinopathies				
Target cells	0.4%	0.84	74.2	93.7
M/H ratio	2.63	0.79	72.5	86.8
Sirdah	33.66	0.66	56.4	88.2
RBC	4.91X10^12^/L	0.64	47.5	88.2
Ehsani	12.8	0.62	67.3	58.8
Discrimination between iron deficiency anemia and thalassemia minor				
Target cells	0.6%	0.88	82.1	90.2

Considering the different subtypes of hemoglobinopathies, there was little or no overlap with IDA for the cases of HbC heterozygosity, HbC homozygosity, HbE disease, sickle cell HbC disease, sickle cell disease, or sickle cell β0‐thalassemia. This was also the case for the 2 patients with thalassemia major. Only 7/51 (13.7%) patients with IDA had more than 0.4% of target cells, and 4 with β‐thalassemia minor (4/28, 14%) had less than this threshold. It was less clear for cases of sickle cell or sickle cell α + β + −thalassemia (Figure S4).

## 5 Discussion

Classical RBC indices do not discriminate patients with microcytosis as suffering from IDA or HbP [19]. This study shows that automated AI‐aided morphological quantification of TC in patients with microcytosis provides a novel, nearly instantaneous and highly specific tool. The two conditions of IDA and HBP can thus be discriminated without the need for any calculation or human microscopic observation. The Mindray integrated instruments allowed using just the primary blood tube, collected on EDTA, for CBC. Upon request by the operator, or automatically if the flags of microcytosis and hypochromia are triggered, the Mindray CAL 8000 2.0 0111 system and MC‐80 can generate, stain, and analyze a blood smear. This analysis takes only 1 min. Quantitative results and images are displayed on the same monitor providing CBC parameters.

Although patients with the genetic anomalies of thalassemia initially originated from the Mediterranean region, Middle East, or parts of Asia [3], population migrations have contributed to the worldwide dissemination of this condition and other HbP. Although IDA is largely the most frequent condition leading to hypochromia and microcytosis, it remains important to properly identify the etiology of these features with a minimum of extra tests. As pointed out by Salinas et al. [4], this may lead to significantly reducing the costs of laboratory investigations and is also likely to have repercussions on the proper management of the patients. Traditionally [5], this type of anemia would lead to collecting a new blood sample for the investigation of IDA. This would mean, at least, a ferritin assay, combined with the assessment of concomitant inflammation by measuring C‐reactive protein [1], but some institutions proceed to a larger exploration of iron transport or even iron stores. For HbP, and sometimes to sort out the etiology of microcytic anemia, Hb electrophoresis and genetic tests have to be carried out. The use of indexes, as summarized in the introduction, would be to orient the type of test to perform, but their validity is still questioned, and they seem quite variable. Here, the comparison of several of them on the same series of patients identified the TC percentage as the most performing, with excellent specificity.

One of the challenges for these indexes is the diagnosis of certain forms of HbP, that is, heterozygous alpha‐thalassemia, frequent in some regions of Europe such as Sardinia, for which these indexes can fail to detect the anomaly.

Morphological identification of RBC anomalies with the CellaVision solution has already been reported to allow for the discrimination of IDA and HbP [20] using TC percentages. Yet lesser specificity and sensitivity were reported, as well as a higher cut‐off of 3.3%, and operator post‐classification [20].

Recently, artificial intelligence (AI) was challenged with the discrimination of IDA and HbP. In the work by Uçucu et al. [21], a first neural network used 13 parameters with the WEKA system. Some of these parameters are not in routine CBC reports. Variations of their “artificial neural network” (ANN) led to draw a receiver operator characteristic (ROC) curve with only CBC parameters (RBC, Hb, hematocrit, MCV, MCH and RDW). The area under the curve (AUC) was 0.987. Another AI application [22] reported RBC and MCV as the major parameters to discriminate between IDA and HBP. These publications [21, 22] are recent yet translate the persisting uncertainty in devising a straightforward method to rapidly discriminate between IDA and HbP in the routine laboratory. Yet these issues are important in order to propose an adequate management of the patients. Transfusion, iron supplementation and resolution of the underlying condition make IDA a curable disorder. Conversely, identifying a thalassemic trait in otherwise asymptomatic individuals has significant consequences for genetic counselling, avoiding the birth of carriers of both abnormal alleles, that is, with thalassemia major. Moreover, iron therapy is not appropriate for patients with HbP who tend to have iron overload [23].

Recently, Chokchaipermpoonphol et al. [24] also used the MC‐80 to examine RBC anomalies in patients screened for thalassemia and also found significantly different percentages of TC between negative (0.15%) and positive (1.02%, *p* < 0.001) patients. However, this did not translate into a significant ROC curve, possibly because the MCV range was broad.

Here, by selecting patients on microcytemia, the difference between patients with IDA and HbP was strikingly significant, making this fast detection of TC a valuable test to discriminate between these two conditions in case of low MCV. Besides the time‐saving AI‐aided identification and enumeration, another added value of the Mindray solution that was applied is the concomitant visual control provided to the operator, allowing for comfortable supervision through the gallery of detected TC. The fact that this study was conducted in a single center in France could be considered a limitation, and multicentric validation could be considered to strengthen these data.

Of course, the TC threshold identified here could also be applied with other methods of target cell evaluation, that is, separate blood analyzer and digital smear imaging in laboratories not equipped with this system. Larger cohorts on different combinations would be useful to confirm the discriminative validity of this investigation.

## Ethics Statement

The samples had been sent to the laboratory for routine complete blood count (CBC) and no specific bloodletting was performed, making patient consent and ethics board authorization unnecessary, as per French law.

## Supporting information


**Figure S1:** Images, counts and percentages of 2642 red cells as displayed by the MC‐80, classified in different types in a case with IDA.
**Figure S2:** Comparative values of hemoglobin (Hb), red blood cells (RBC), mean corpuscular hemoglobin concentration (MCHC), mean corpuscular volume (MCV) and microcytes between patients with iron deficiency anemia (IDA, *n* = 51) or hemoglobinopathies (HbP, *n* = 101).
**Figure S3:** Grouped information of parameter distribution and ROC curves for target cells between IDA and hemoglobinopathies, with a focus on β‐thalassemia and target cells. (See also Table 2). Top row: left comparison of IDA and β‐thalassemia; right M/H ratios of IDA and HBP. Second row: Roc curves for the discrimination of IDA versus HBP by target cell percentages; IDA versus β‐thalassemia by target cell percentages; IDA versus HBP by M/H ratio. Third row: Absence of discrimination between IDA and HBP using Sirdah index, red blood cell counts and Eshani index. Fourth row: Poor results of ROC curves for the discrimination between IDA and HBP using Sirdah index, red blood cell counts and Eshani index.
**Figure S4:** Percentages of target cells according to IDA or hemoglobinopathy types.

## Data Availability

The data that support the findings of this study are available from the corresponding author upon reasonable request.
