# Supplementary material for: Automated Morphologic Differentiation Between Iron Deficiency Anemia and Thalassemia
Source: J Clin Lab Anal. 2025 Sep 3;39(19):e70097. doi: 10.1002/jcla.70097 (PMC12514970; doi:10.1002/jcla.70097)

**Automated morphologic differentiation between iron deficiency anemia and thalassemia**

Julien Guy^1^, Marie-C Béné^2^, Ramon Simon Lopez^3^_,_ Marc Maynadié^1^_,_ Céline Row ^1^

^1^Hematology Biology, Dijon University Hospital, Dijon, France

^2^Hematology, Nantes University, Nantes, France

^3^Mindray, Scientific Research. Oberwil bei Zug, Switzerland

Supplementary material

**Supplementary Figure S1.** Images, counts and percentages of 2642 red cells as displayed by the MC-80®, classified in different types in a case with IDA


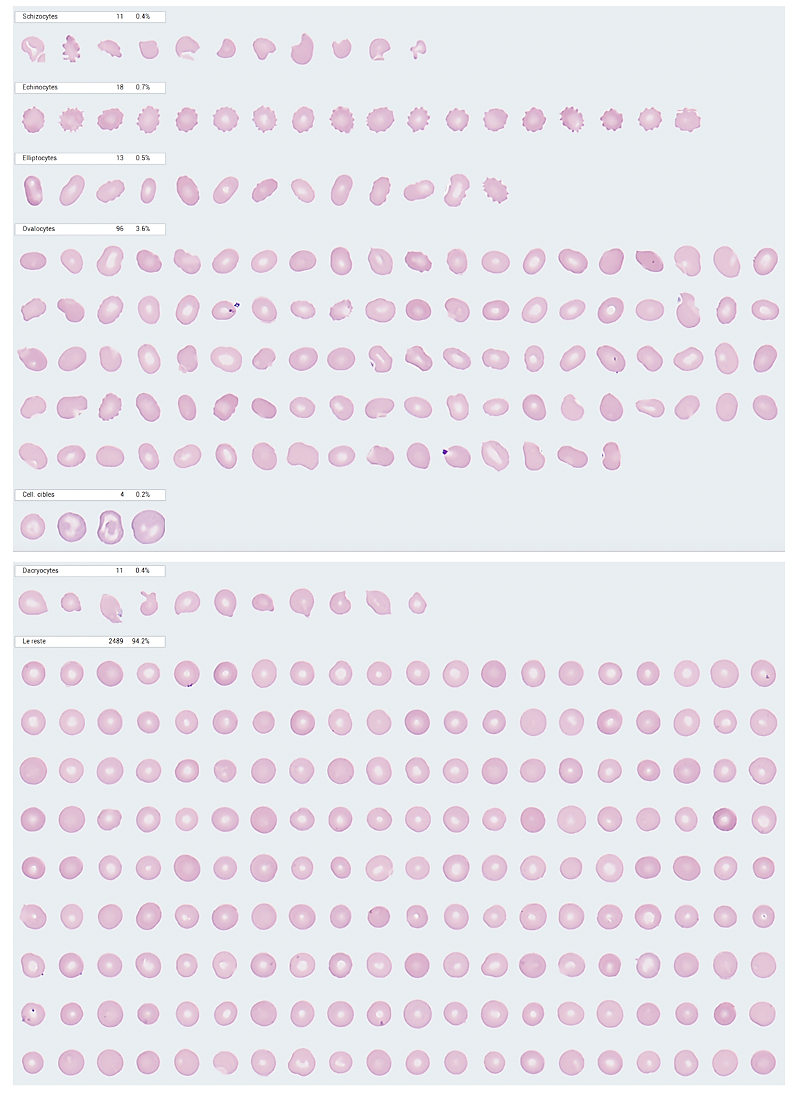


Schistocytes n=11, 0.4%

Echinocytes n=18, 0.7%

Elliptocytes n=13, 0.5%

Ovalocytes n=96, 3.6%

Target cells n=4, 0.2%

Dacryocytes n=11, 0.4%

Other RBC n=2489, 94.2%

**Supplementary Figure S2.** Comparative values of hemoglobin (Hb), red blood cells (RBC), mean corpuscular hemoglobin concentration (MCHC), mean corpuscular volume (MCV) and microcytes between patients with iron deficiency anemia (IDA, n=51) or hemoglobinopathies (HbP, n=101).


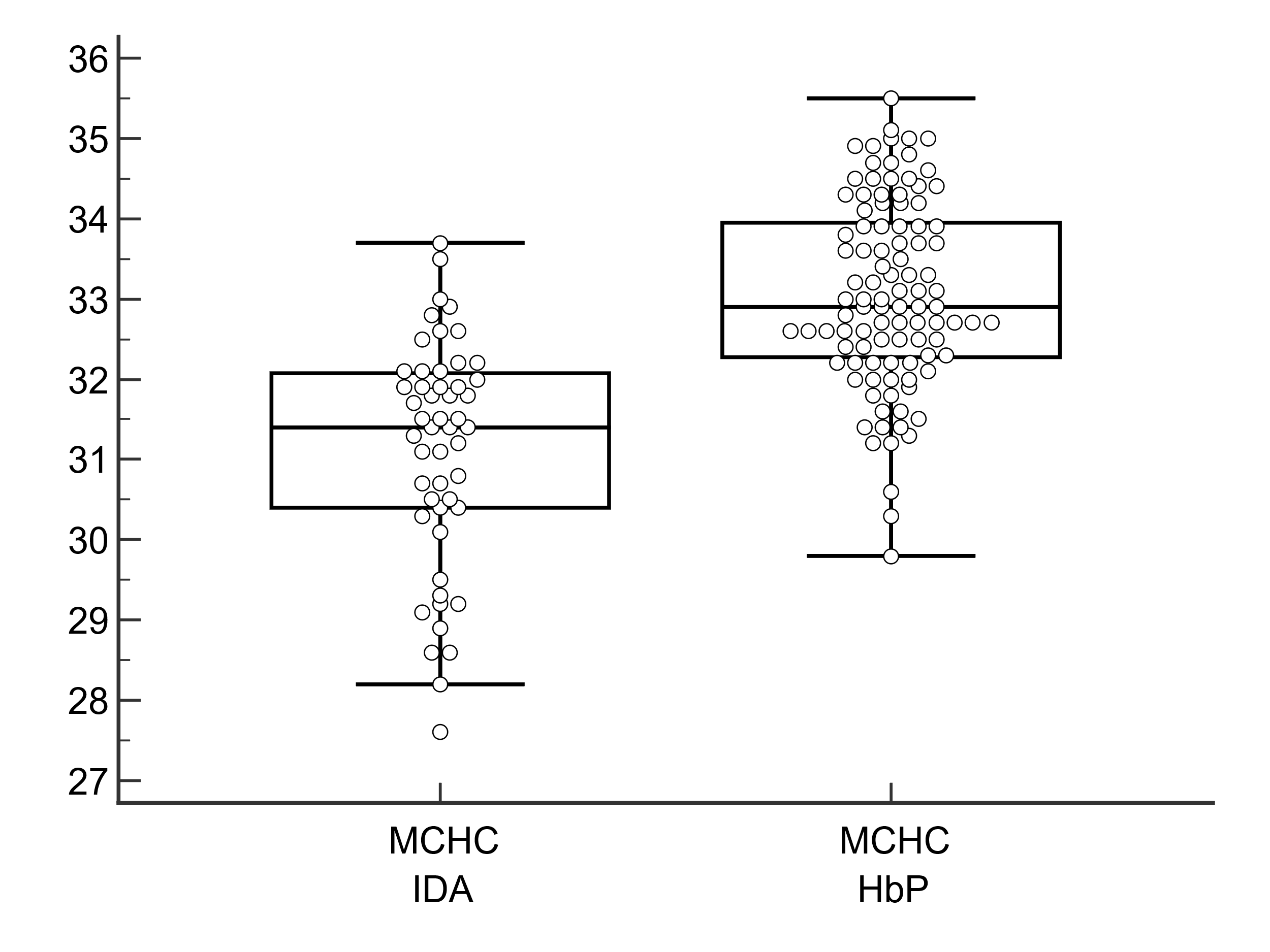

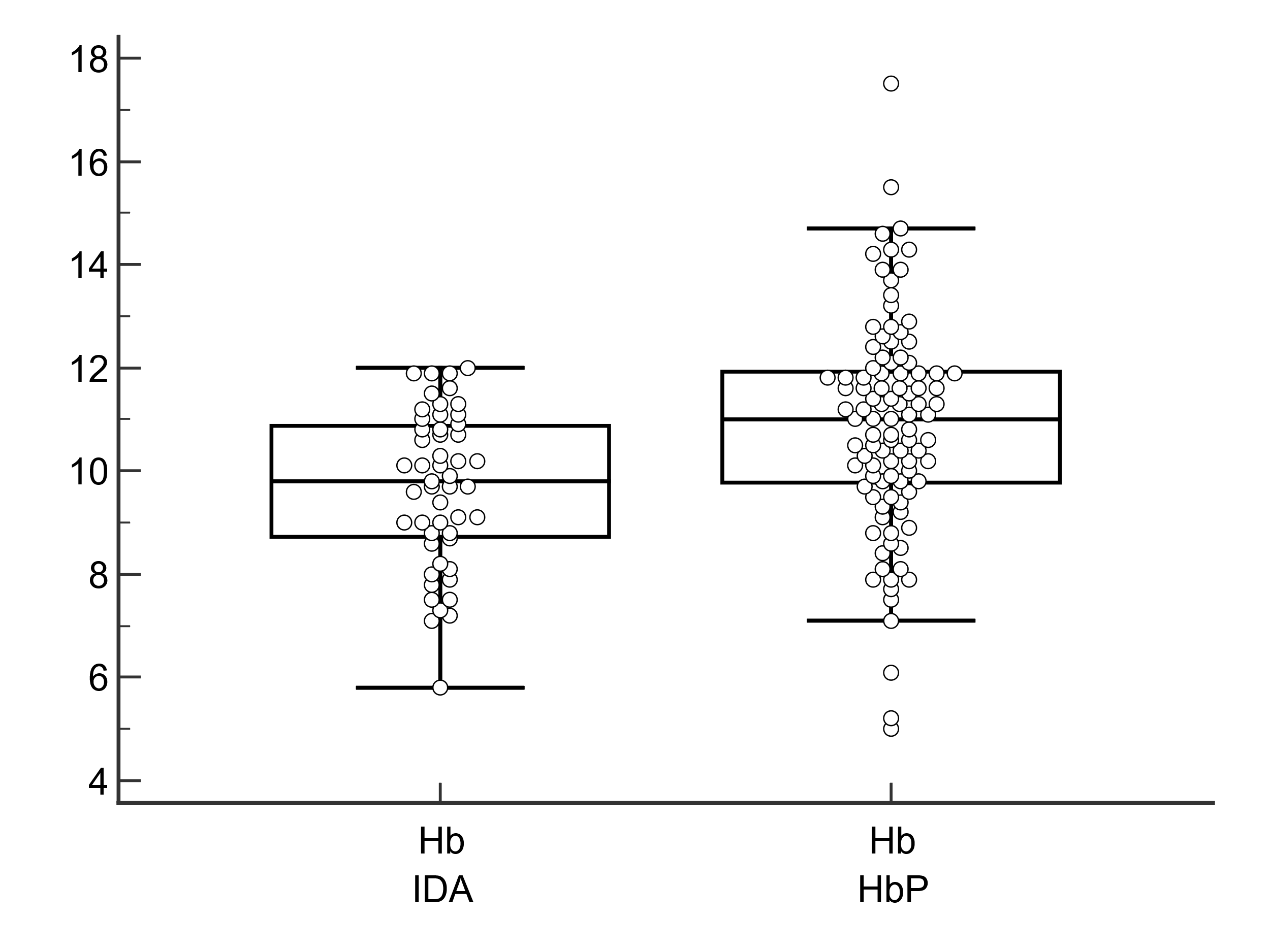

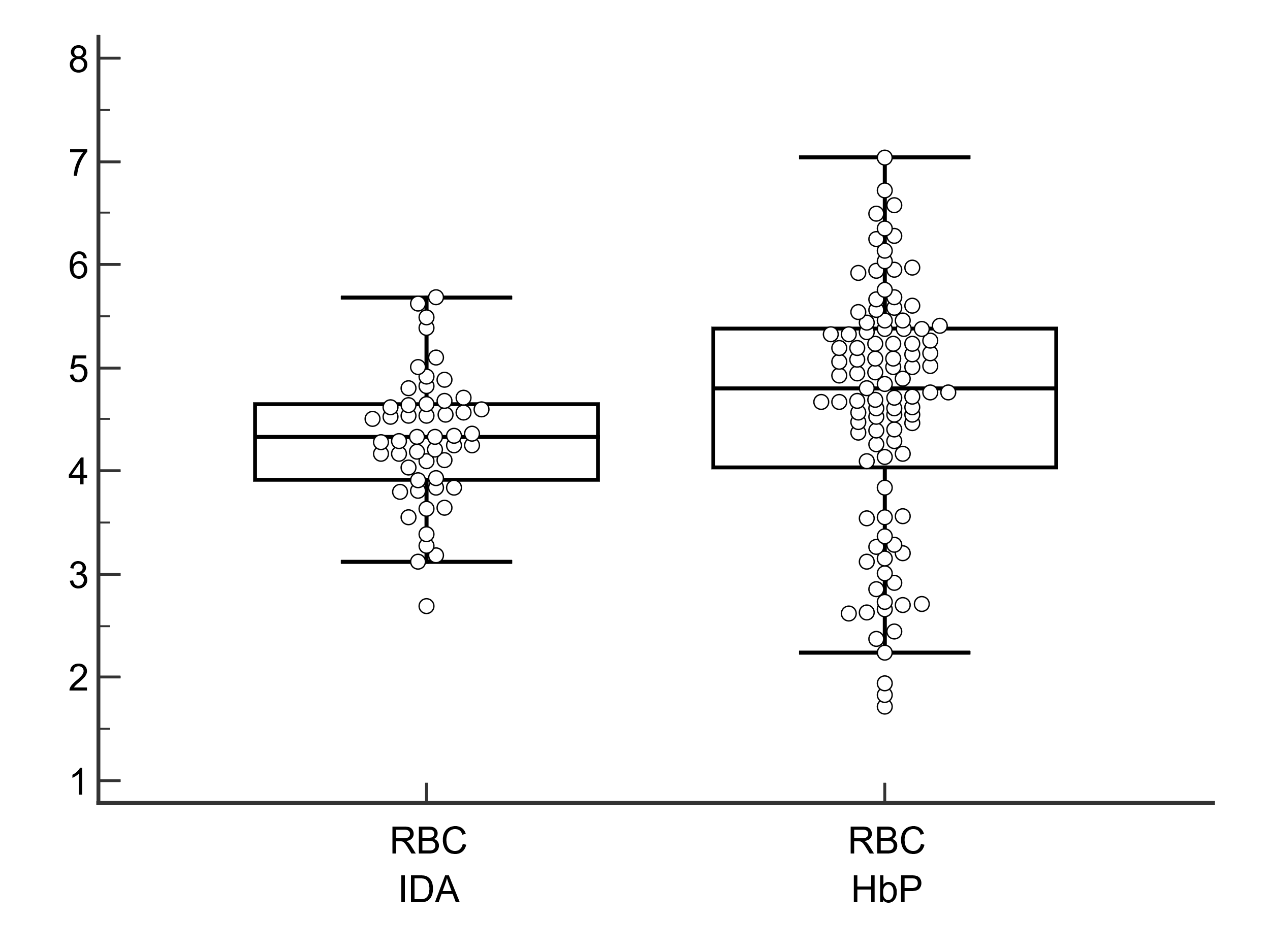

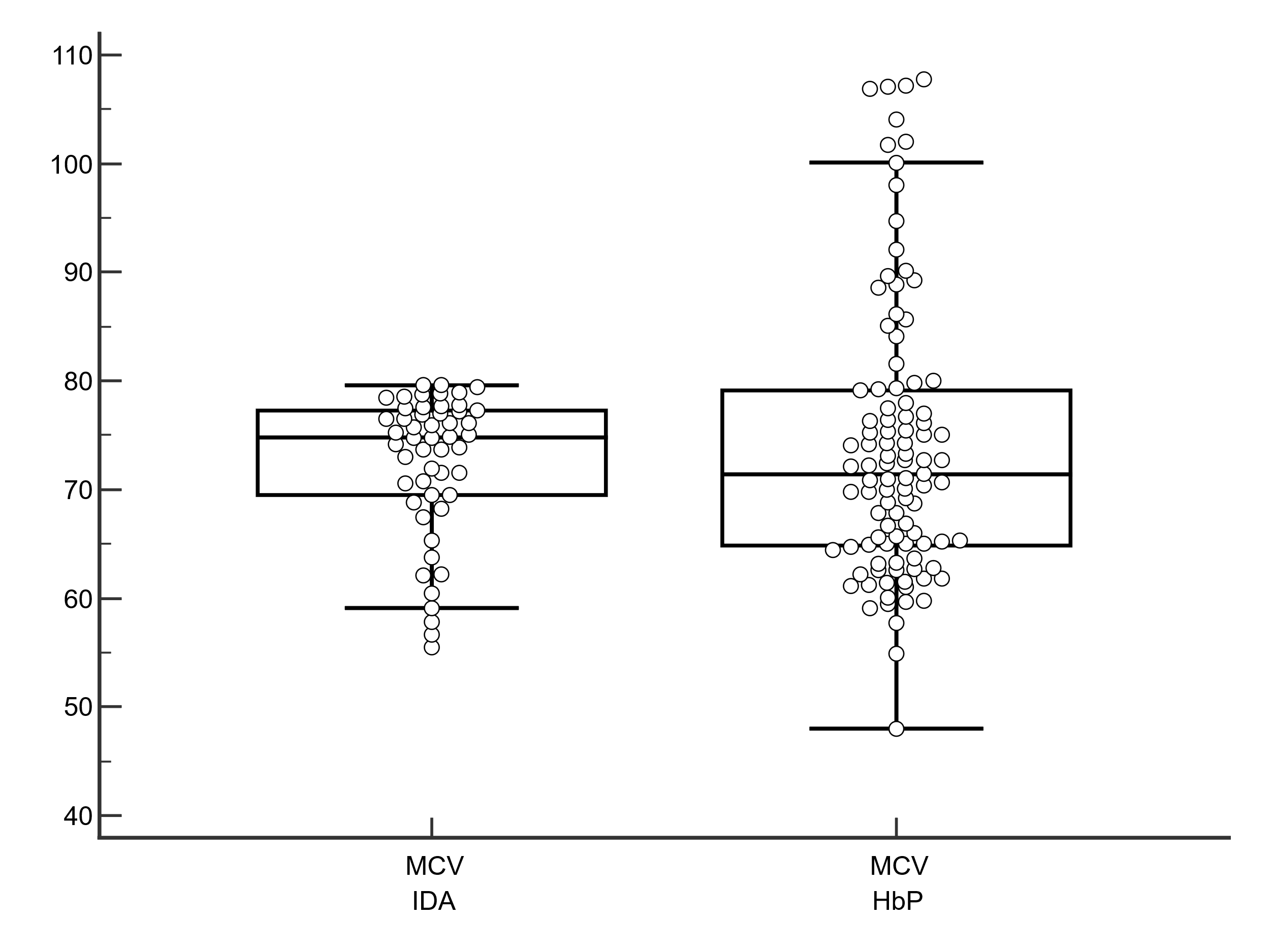

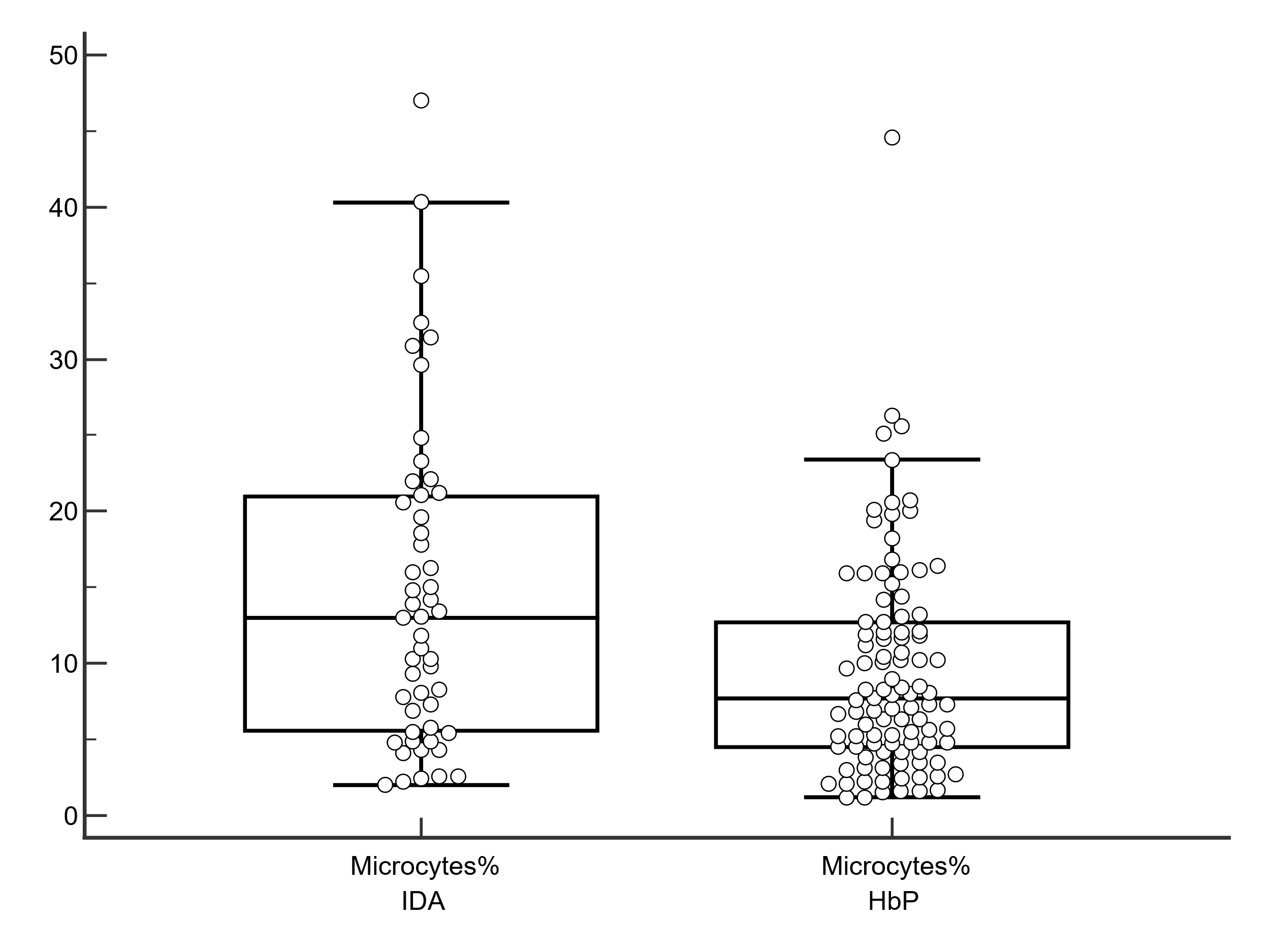


g/dL

X10^9^/L

g/dL

fL

%

**Supplementary Figure S3**. Grouped information of parameter distribution and ROC curves for target cells between IDA and hemoglobinopathies, with a focus on β-thalassemia and target cells. (See also table 2).

Top row: left comparison of IDA and β-thalassemia; right M/H ratios of IDA and HBP

Second row: Roc curves for the discrimination of IDA vs. HBP by target cell percentages; IDA vs. β-thalassemia by target cell percentages; IDA vs. HBP by M/H ratio.

Third row: Absence of discrimination between IDA and HBP using Sirdah index, red blood cell counts and Eshani index.

Fourth row: Poor results of ROC curves for the discrimination between IDA and HBP using Sirdah index, red blood cell counts and Eshani index.


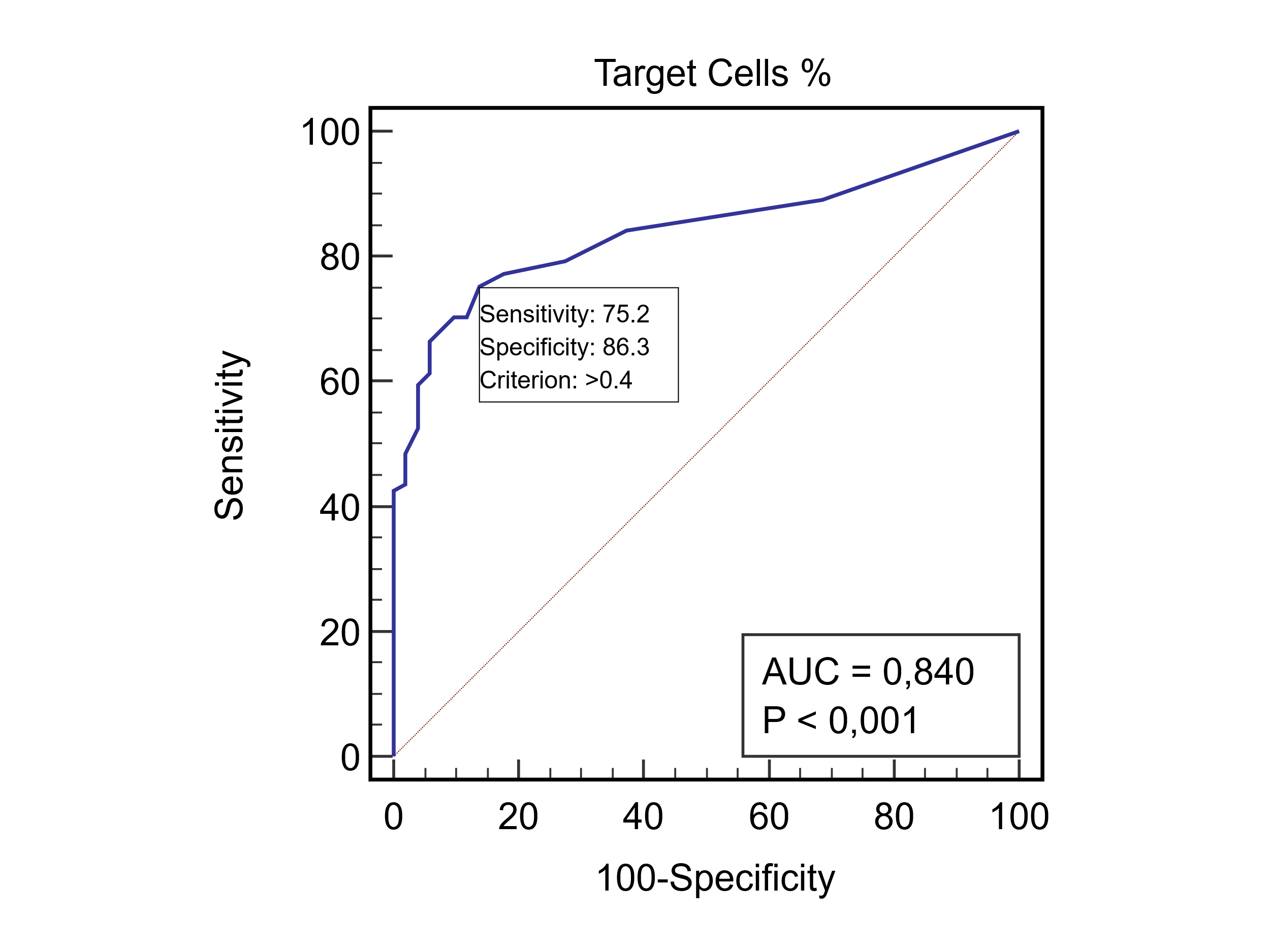

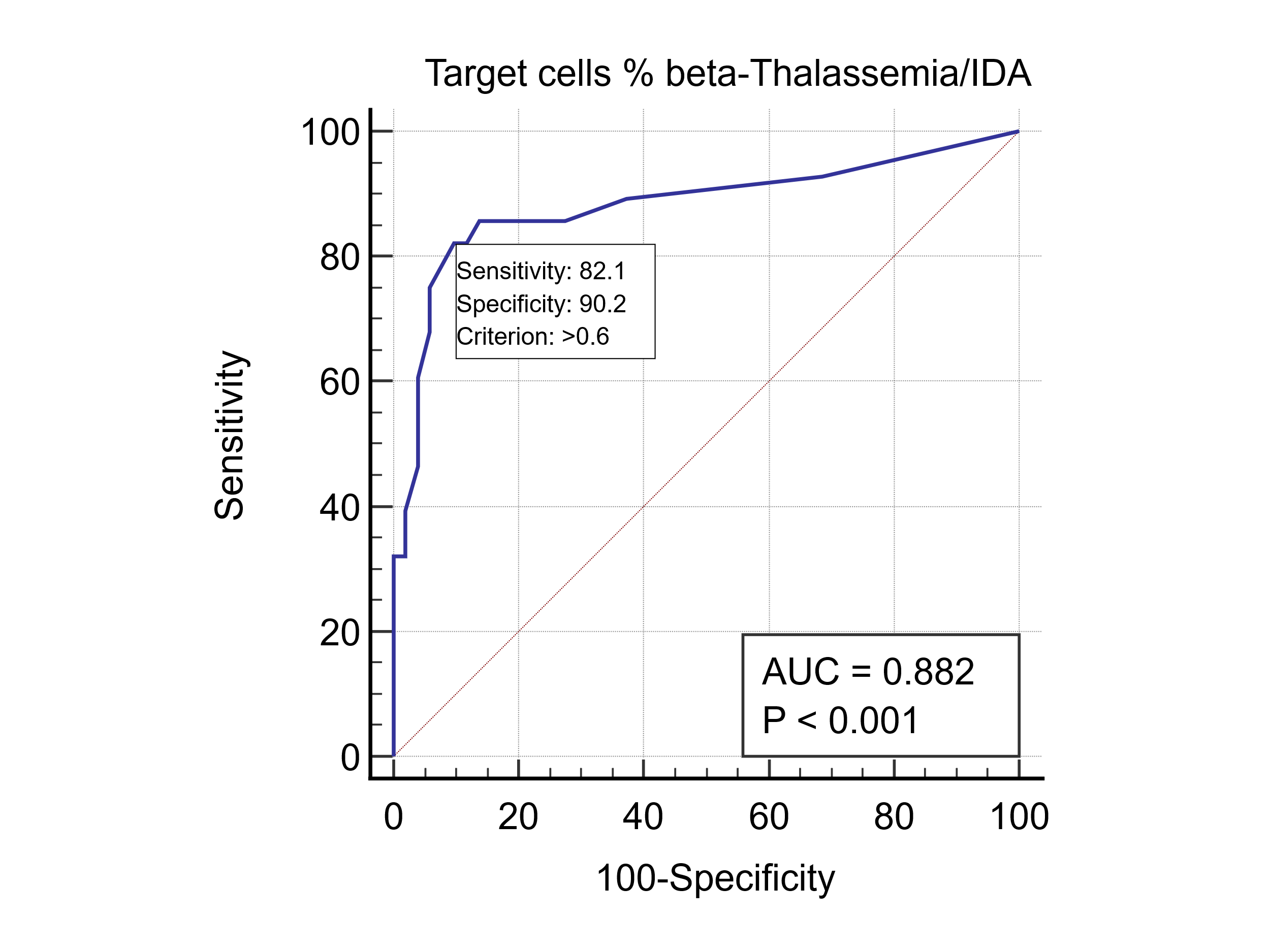

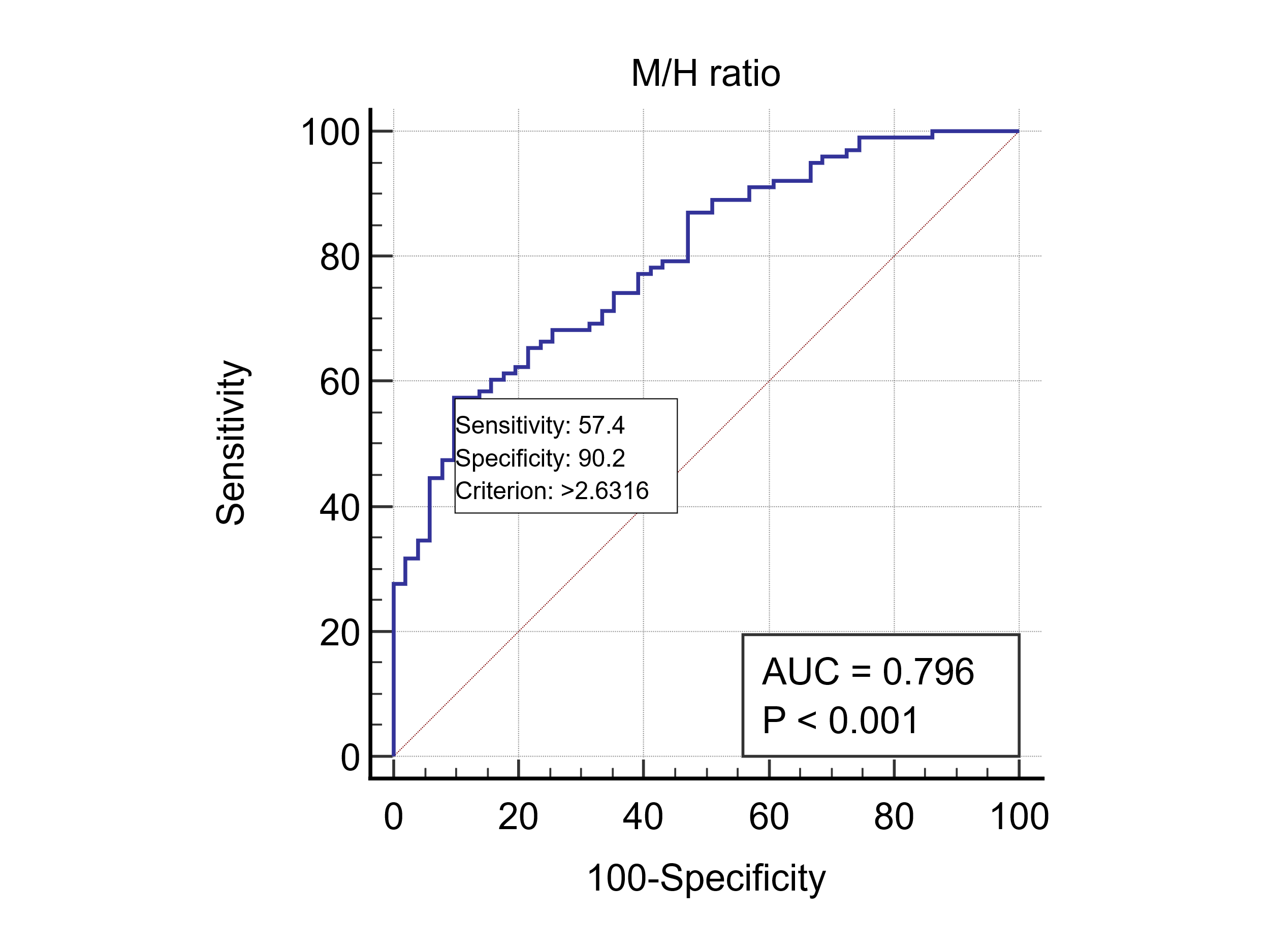

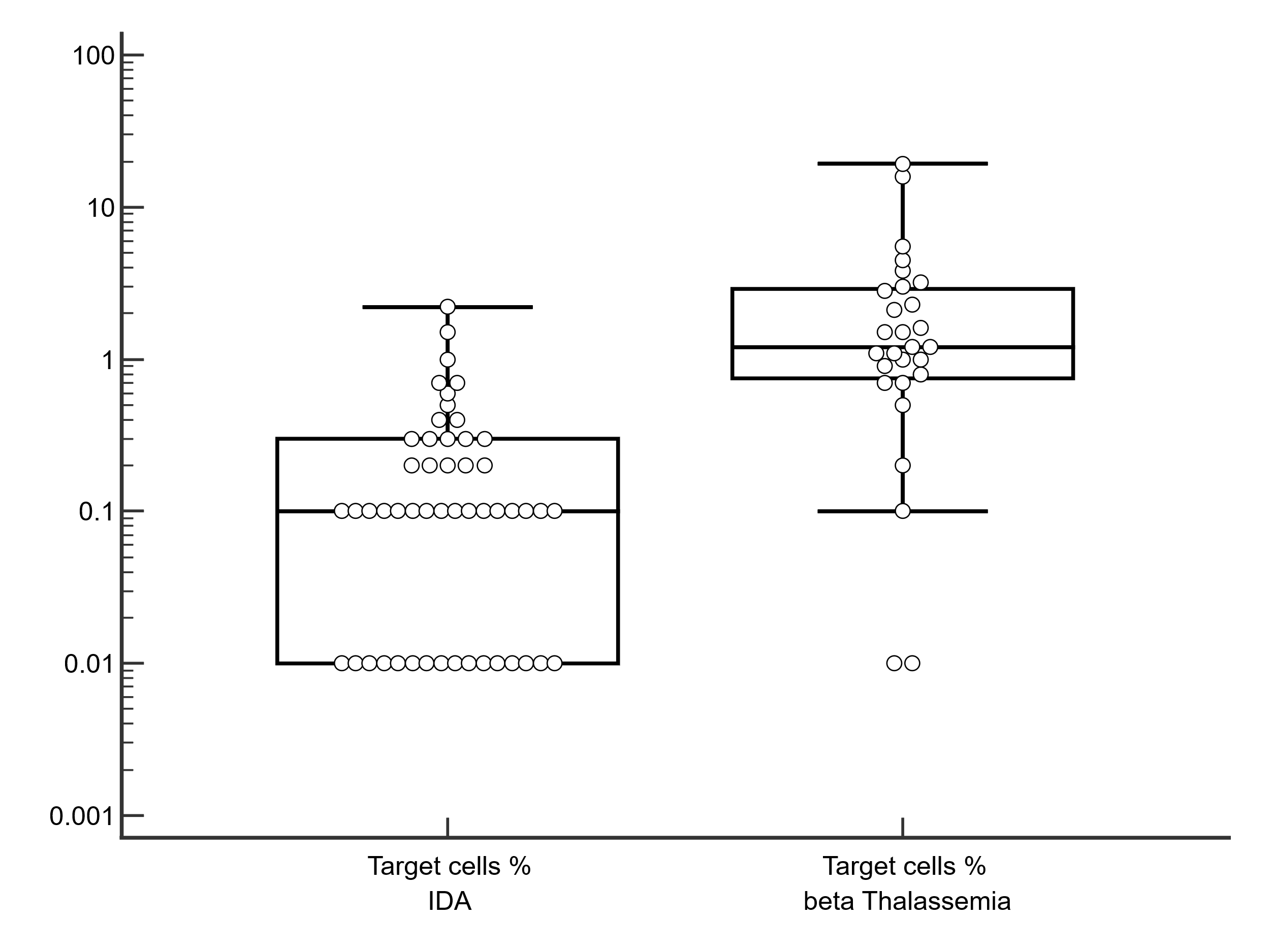

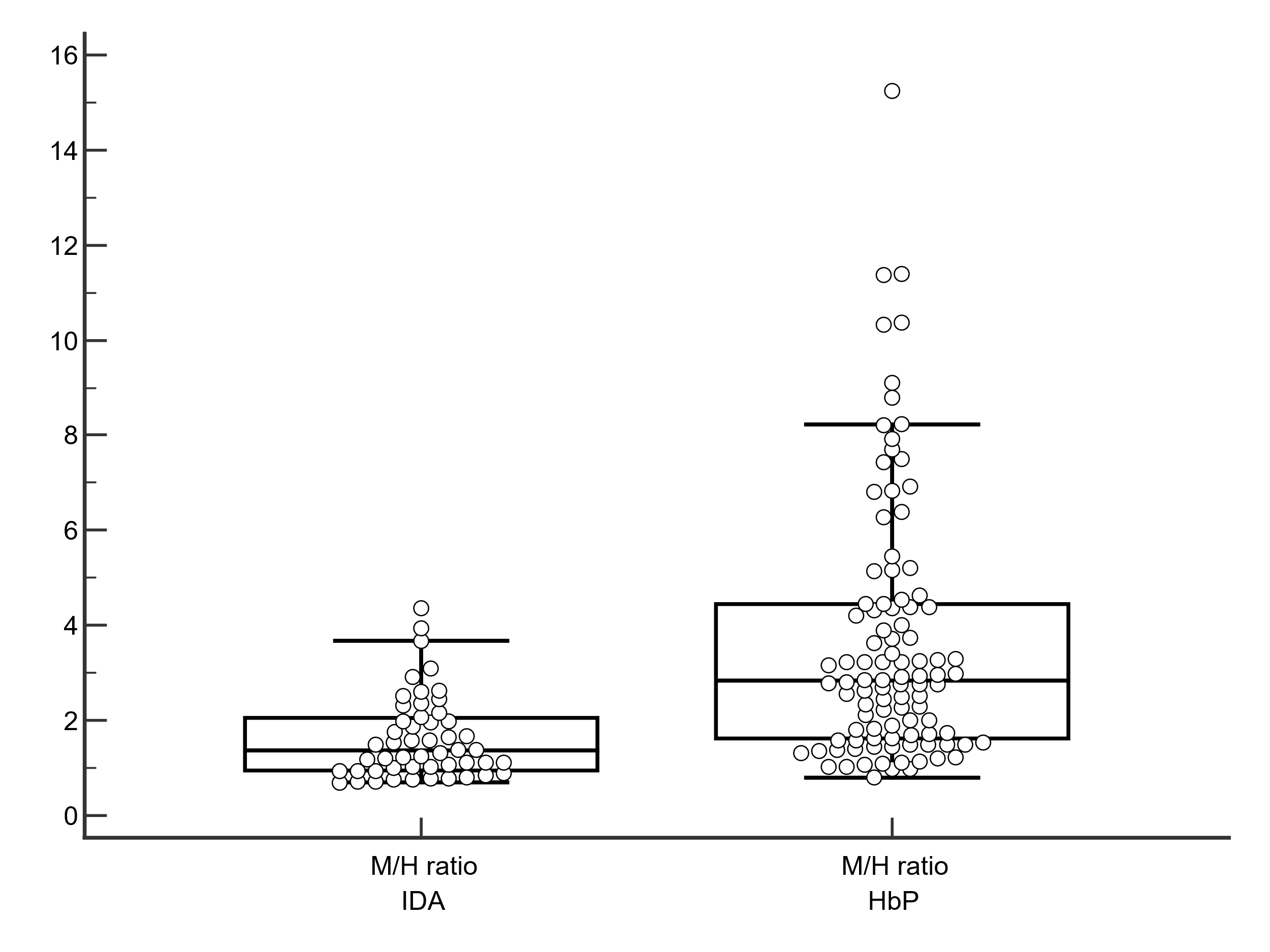

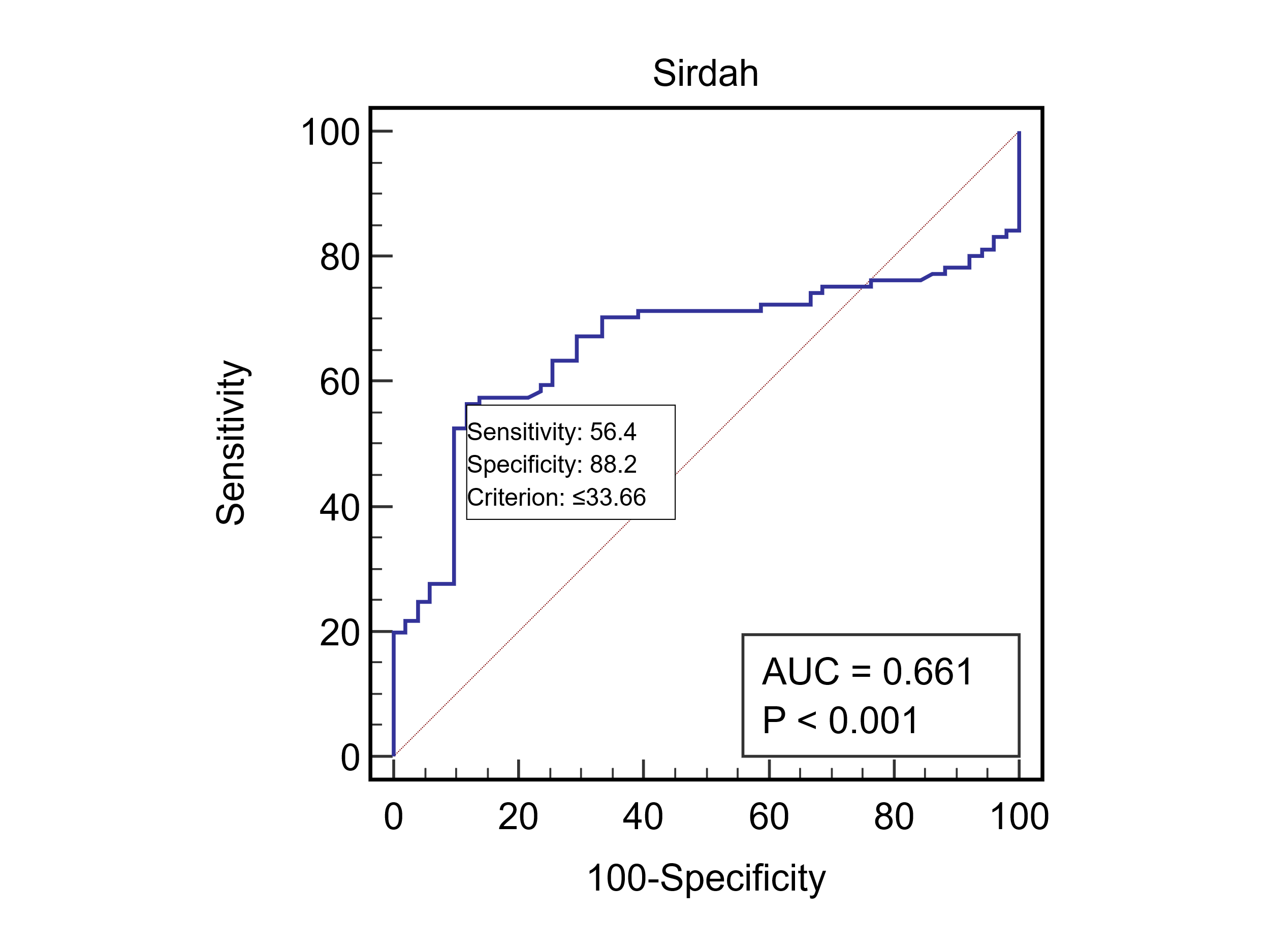

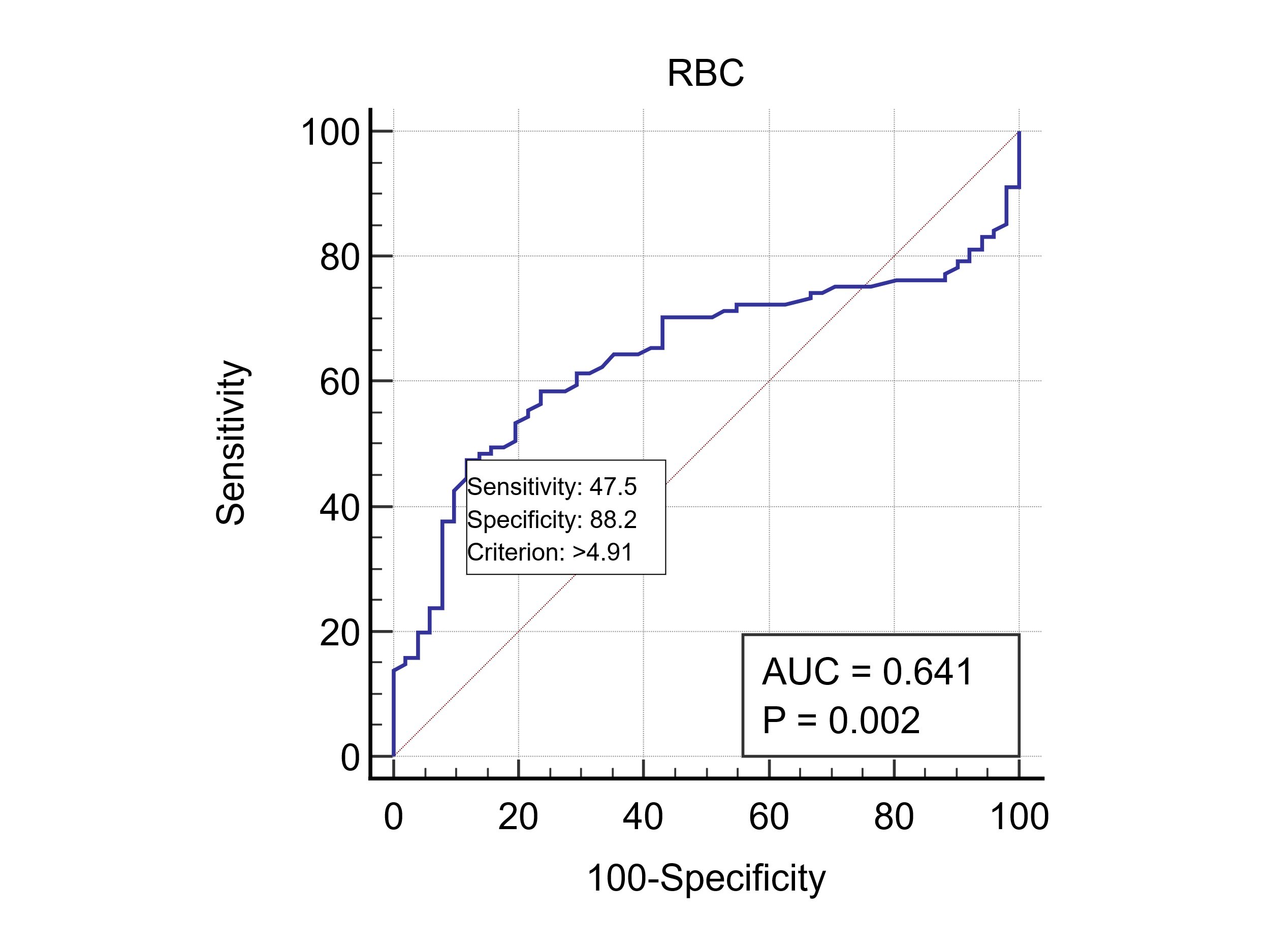

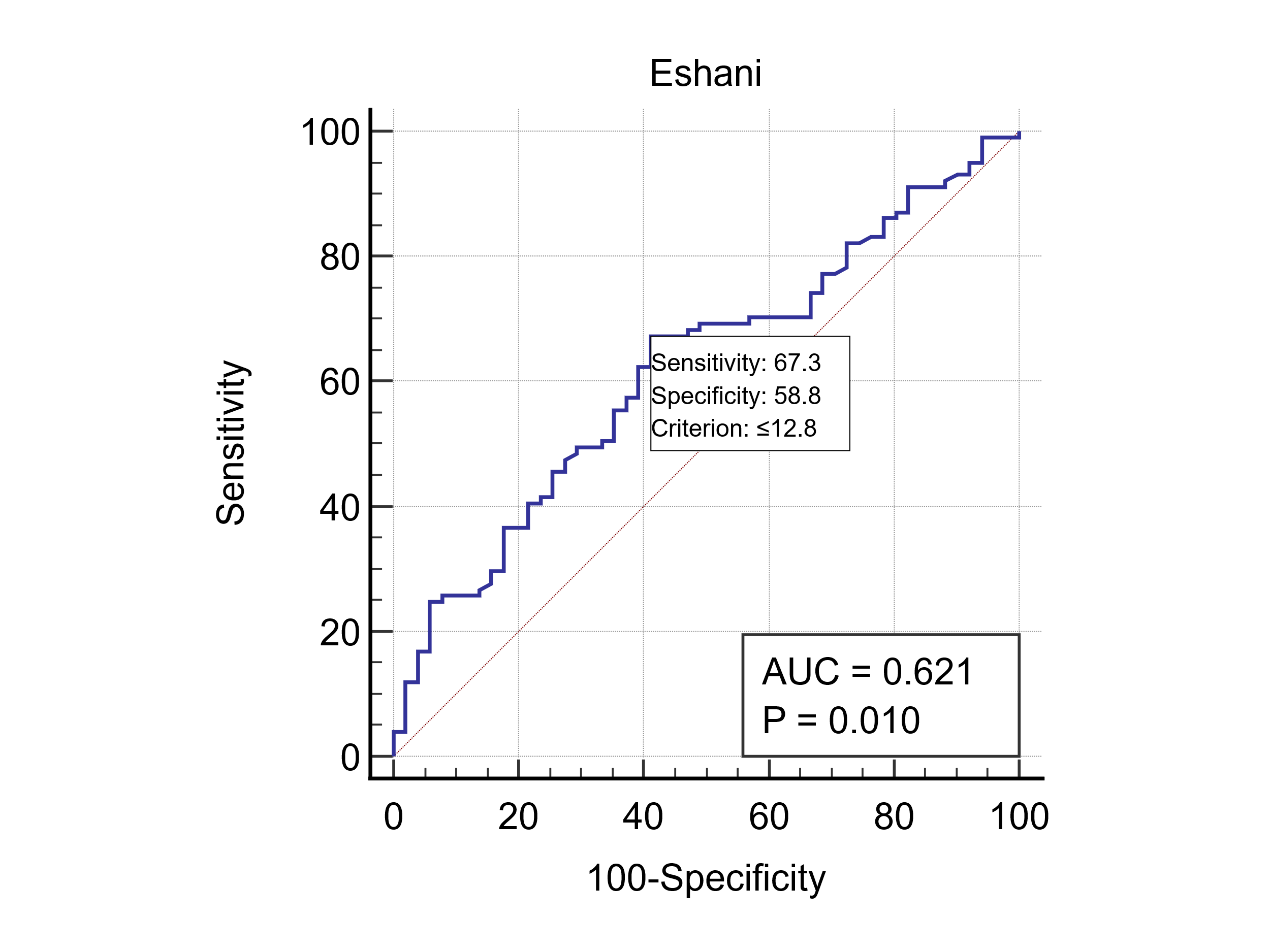

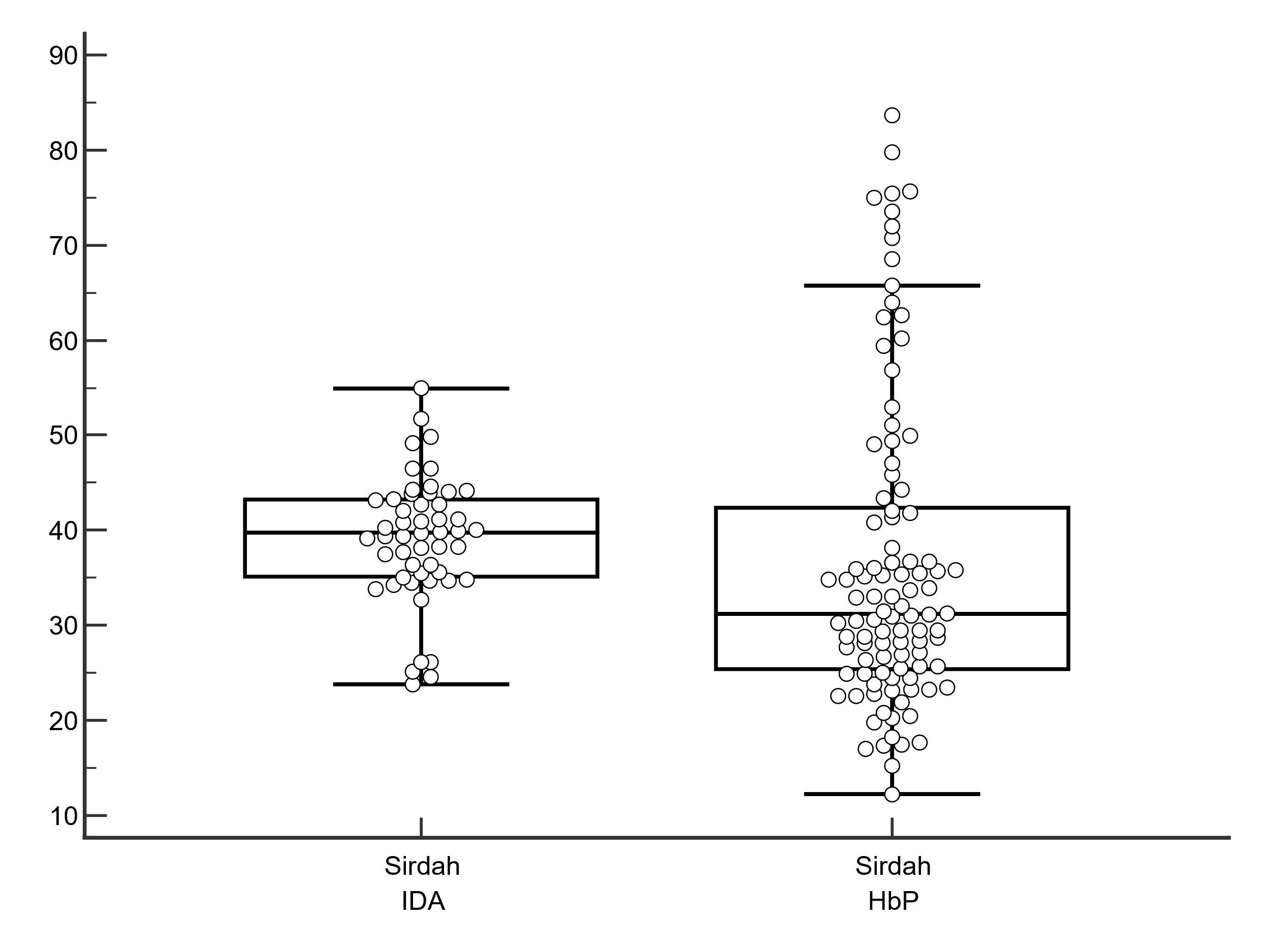

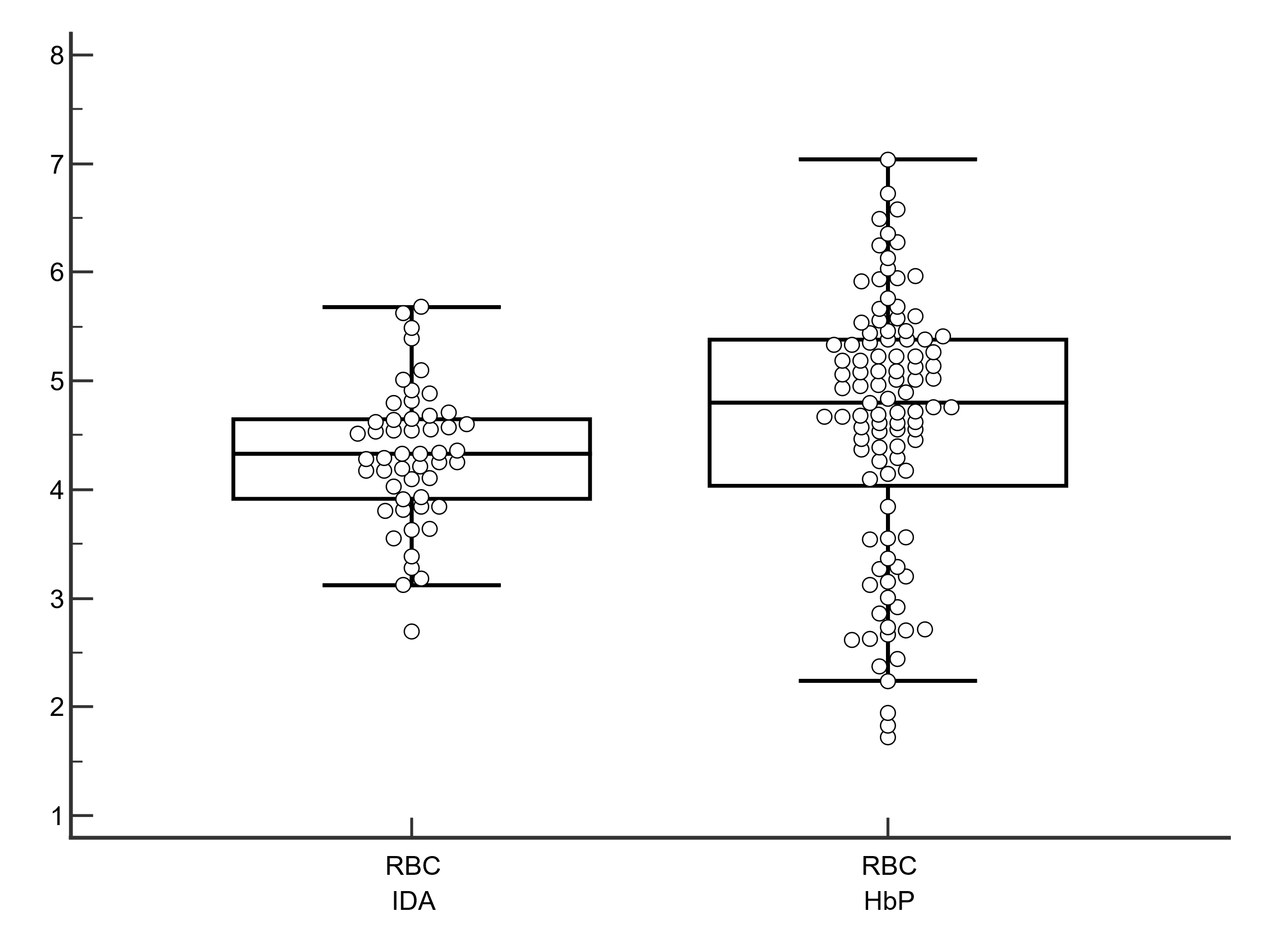

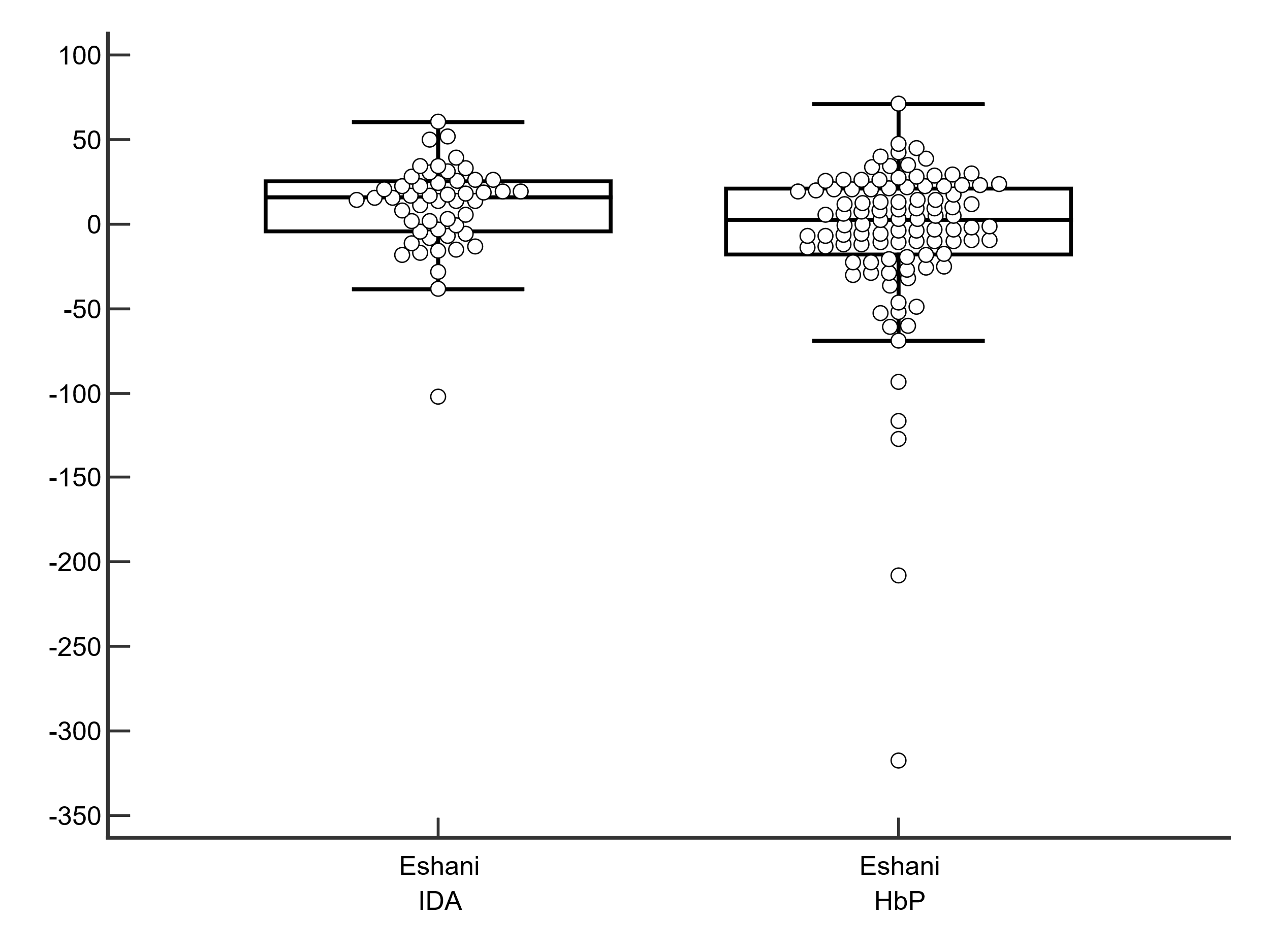


**Supplementary Figure S4**. Percentages of target cells according to IDA or hemoglobinopathy types.


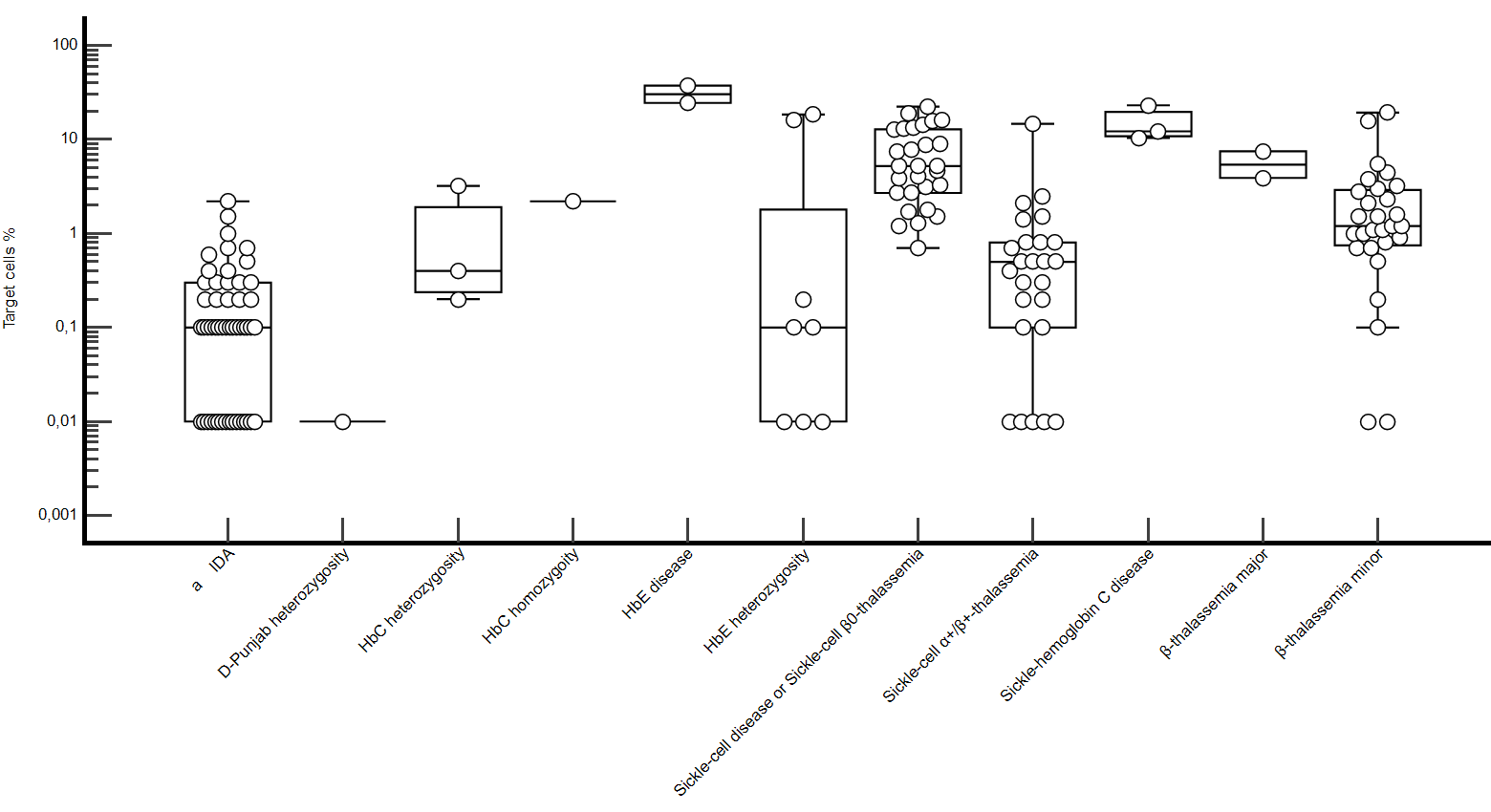

Supplement: Supplementary file 1 — Figure S1: Images, counts and percentages of 2642 red cells as displayed by the MC‐80, classified in different types in a case with IDA. Figure S2: Comparative values of hemoglobin (Hb), red blood cells (RBC), mean corpuscular hemoglobin concentration (MCHC), mean corpuscular volume (MCV) and microcytes between patients with iron deficiency anemia (IDA, n = 51) or hemoglobinopathies (HbP, n = 101). Figure S3: Grouped information of parameter distribution and ROC curves for target cells between IDA and hemoglobinopathies, with a focus on β‐thalassemia and target cells. (See also Table 2). Top row: left comparison of IDA and β‐thalassemia; right M/H ratios of IDA and HBP. Second row: Roc curves for the discrimination of IDA versus HBP by target cell percentages; IDA versus β‐thalassemia by target cell percentages; IDA versus HBP by M/H ratio. Third row: Absence of discrimination between IDA and HBP using Sirdah index, red blood cell counts and Eshani index. Fourth row: Poor results of ROC curves for the discrimination between IDA and HBP using Sirdah index, red blood cell counts and Eshani index. Figure S4: Percentages of target cells according to IDA or hemoglobinopathy types. [file JCLA-39-e70097-s001.docx]
